# Life Satisfaction as Related to Personal Values and Self-Evaluation Dimensions in a Greek Adult Population

**DOI:** 10.3390/bs16071083

**Published:** 2026-07-01

**Authors:** Aikaterini Gari, Kostas Mylonas

**Affiliations:** Department of Psychology, School of Philosophy, National and Kapodistrian University of Athens, Panepistimiopolis, Ilissia, 15784 Athens, Greece; kmylonas@psych.uoa.gr

**Keywords:** life satisfaction, personal values, general self-efficacy, personality dispositions, religiosity, mediation

## Abstract

This study investigates life satisfaction in Greek adults and its association with personal values (PVQ-RR), religiosity, and self-evaluation dimensions, such as general self-efficacy, metacognitive dimensions of general self-efficacy, and four personality dispositions following Rohner’s Interpersonal Acceptance–Rejection Theory (IPARTheory). Data were collected online (N = 706 adults, 72.4% females). The mean age was 28.27 (SD = 12.06), while 74.10% of the participants reported currently staying in urban locations; 66.3% were students, and 43% were married or in a relationship. The main aims were to: (a) identify the structure of personal values in conjunction with religiosity, self-evaluation measures, personality dispositions (negative self-esteem, negative self-adequacy, emotional instability, and negative worldview), and life satisfaction and (b) examine the causal pathways from self-evaluation measures and religiosity to life satisfaction, mediated by higher-order personal values and co-existing personality dispositions. Confirmatory factor analysis supported the structure for the higher-order personal value dimensions—Self-Transcendence, Self-Enhancement, Openness to Change, and Conservation—as combined with single constructs for personality dispositions and self-evaluation. Further results indicated a small partial mediating effect of the higher-order values of Self-Transcendence and Self-Enhancement on the relationship between general self-efficacy and life satisfaction. A religiosity–life satisfaction relation was also mediated through the Self-Transcendence higher-order value.

## 1. Introduction

Subjective well-being represents a higher-order construct that includes both happiness and Diener’s life satisfaction (Diener et al., 2013). Happiness, as a central construct in psychological research, was defined as a form of subjective well-being that serves as a fundamental indicator of individuals’ quality of life (Lu & Gilmour, 2004). With respect to Diener’s approach to satisfaction with life, its operational definition regards a type of happiness that enhances one’s optimistic life view (Diener et al., 1985). A review (Diener, 1984) on subjective well-being and satisfaction with life defined the latter as a cognitive and judgmental process that frames a person-oriented satisfaction with aspects of the European–American cultural contexts and also emphasizes the standards individuals adopt or perceive as unrelated to external factors (Diener et al., 1985). Therefore, Diener’s life satisfaction is a part of subjective well-being, including people’s judgments of how satisfied they are with their present state of affairs as compared with the standards each individual sets for himself/herself. These theoretical distinctions have been incorporated into the manuscript, ultimately leading to a focus on the single construct relevant to the purpose of the present study, namely, life satisfaction. The Scale for Satisfaction with Life (SWLS) he developed asks participants to appraise their general life satisfaction, emphasizing the cognitive component of subjective well-being. This scale demonstrated favorable psychometric properties, and it has been established as a fundamental and user-friendly instrument for assessing life satisfaction.

Cross-cultural studies dissociate individual life satisfaction and interdependent satisfaction. The latter represents the degree to which an individual is connected harmoniously with the collective way of well-being (Krys et al., 2019) or with group happiness. It is conceptualized as an idealized group happiness that is highly scored by East Asian cultures such as the Japanese, Korean, and Chinese cultural settings (Diener et al., 1985). However, another study (Krys et al., 2023) concluded that both highly collectivistic countries and individualistic countries (such as Canada and the United States) would score lower on individual happiness than on family happiness, in alignment with the principle that family is the “fundamental team” of survival across cultures.

Later on, several types of data indicated that the SWLS validly reflects the quality of respondents’ lives, with respect to objective conditions of specific countries, differences between groups within a cultural setting, genetic and physiological conditions in participants’ lives, and systematic patterns of change before, during, and after significant life events (Diener et al., 2013). In this study, life satisfaction is explored in an adult population in Greece in association with personal values based on the PVQ-RR scale (Schwartz et al., 2017), four personality dispositions that may contribute to adjustment in various circumstances (Rohner, 2004, 2005), dimensions of self-evaluation in terms of self-efficacy types, and one’s religiosity.

Life satisfaction may be linked to individuals’ value orientations through indirect pathways (Diener et al., 2013). Values as general enduring beliefs precede and regulate behaviors via motivation processes (Gari et al., 2005; Rokeach, 1973; Schwartz, 1992; Tatarko et al., 2020). Optimistic or pessimistic orientations of behavior and beliefs for high or low life satisfaction may implicitly be motivated by one’s personal values. The motivational value types delineated in Schwartz’s theory have been empirically identified across and within cultures and have subsequently been used to conceptualize value priorities. This universally valid theory of basic human values first included 10 distinct personal values that were presumed to encompass the major value orientations recognized universally (Schwartz, 2006; Sagiv & Schwartz, 2000). Values functioning as criteria for judging other people and social events are defined as broad motivational goals that transcend specific situations, guide decisions, and moderate behavior; they are also closely associated with emotions rather than with objective and factual judgments. Thus, each of the ten values of the Schwartz model is defined by the goals towards which it is directed and the motivation it expresses (Schwartz, 1992; Bilsky & Schwartz, 1994). Portrayed on a schematic circular structure represents a continuum of related motivations through patterns of value relations that express either conflict or congruity, e.g., the more distant two values appear around the circle, the more antagonistic their underlying motivations are (Sagiv & Schwartz, 2000; Schwartz, 1992, 2006). Consequently, human values are ultimately differentiated by the priorities and levels of importance that individuals assign to them (Schwartz et al., 2012).

Four higher-order value dimensions capture the principal contrasts among competing value orientations. The dimension of *Openness to Change* versus *Conservation* reflects the tension between values that emphasize the autonomous, self-expressive experience along with independence of thought, action, feeling, and readiness for change; Conservation emphasizes values that prioritize self-restriction, order, and resistance to change. The second dimension, *Self-Enhancement* versus *Self-Transcendence*, represents the conflict between values oriented towards the pursuit of one’s own interests, relative success, and dominance versus those that emphasize concern for the welfare and interests of others and nature. Later on, Schwartz’s revised theory included 19 values separating the *person-focused values* and the *social-focused values* (Schwartz & Butenko, 2014; Schwartz et al., 2012). The person-focused values primarily regulate how one expresses one’s own personal characteristics and interests and include two out of four higher-order values, Self-Enhancement and Openness to Change. The social-focused values primarily regulate how one relates socially to others and preserves cooperative relations; this dimension includes Conservation and Self-Transcendence (Schwartz, 2006; Sortheix & Schwartz, 2017). Finally, on the basis of the way values are related to anxiety, values are also organized in terms of anxiety control; they are based either *on the need to avoid* or *on the need to control anxiety and threat* in order to protect the self.

The focus of our exploration was the mediative association that the PVQ-RR’s four higher-order personal values may have with the relationship between personality dispositions, religiosity, self-evaluations perceived as general self-efficacy and metacognitive general self-efficacy, and sense of life satisfaction. Such a multidimensional system of factors that may influence life satisfaction either directly or through mediating mechanisms is inherently dynamic and, therefore, not readily amenable to accurate *a priori* predictions. The international literature does not provide definitive conclusions regarding these complex relationships; rather, it has primarily examined specific pairs of associations or interconnections of values involving life satisfaction. Consequently, the investigation of these relationships through direct effects and mediation paths constitutes a key element of the originality and contribution of the present study.

In reference to the interconnections between personal values and life satisfaction, it has been suggested that pursuing growth values such as self-direction, benevolence, universalism, achievement, and stimulation tends to promote well-being because they are self-actualizing and self-growth-oriented normative beliefs. However, values such as conformity, tradition, security, and power tend to undermine well-being because they reflect the need to protect oneself against insecurity and threat (Bilsky & Schwartz, 1994; Sagiv & Schwartz, 2000). Values that are directed toward intrinsic goals of relatedness, autonomy, and competence are expected to be positively related to subjective well-being, but values directed toward extrinsic goals of wealth, fame, power, and attractiveness are negatively related (Deci & Ryan, 2000; Kasser et al., 2004). Other cross-cultural studies also reported correlations between values and subjective life satisfaction (Bobowik et al., 2011; Cohen & Shamai, 2009; Joshanloo & Ghaedi, 2009; Sagiv & Schwartz, 2000; Sortheix & Lönnqvist, 2014; Sortheix & Schwartz, 2017).

Life satisfaction grounded in Diener’s conceptualization has been consistently found to correlate positively and moderately with general self-efficacy, a distinct term that has been theorized by Bandura (1977, 1997) as a set of individuals’ beliefs about their capacity to manage challenges and achieve desired outcomes. It has been generally associated with adaptive functioning and well-being, including life satisfaction, across various contexts (Luszczynska et al., 2005). Empirical studies showed that general self-efficacy is one of the strongest correlates of life satisfaction for university students (Azizli et al., 2015), a meaningful predictor for young adults (Çakar, 2012), and for married women (Poorbaferani et al., 2018). A recent study using structural equation modeling extended the effect of self-efficacy to life satisfaction and highlighted one’s career-related perceptions as an indirect pathway (Gerçek & Özveren, 2026). In addition to Bandura’s contribution on self-efficacy, researchers became interested in the more trait-like general dimension of self-efficacy, which has been termed “general self-efficacy” (GSE) (e.g., Gardner & Pierce, 1998; Judge et al., 2000); it has been defined as an individual’s generalized belief in one’s competence to organize and execute the actions required to attain desired outcomes across diverse situations. GSE has been shown to be positively related to learning goal orientation and other motivational traits, including the need for achievement and conscientiousness (Chen et al., 2000). For this study, GSE was assessed via the New General Self-Efficacy scale (NGSE), which exhibits favorable psychometric properties, and it has been successfully correlated with life satisfaction (Chen et al., 2001).

Bandura’s model on self-efficacy also included some metacognitive forms (Bandura, 1997) that are not recognized via a concrete term, such as metacognitive self-efficacy, but under closely related formulations, such as self-efficacy for self-regulated learning, metacognitive efficacy beliefs, or self-efficacy for cognitive regulation (Coutinho, 2007; Pajares, 2007; Schraw & Dennison, 1994; Zimmerman, 2000, 2002; Zimmerman & Moylan, 2009). It refers to individuals’ beliefs in their capability to regulate and control their cognitive processes during learning, including planning, monitoring, and evaluating their understanding (Bandura, 1997; Zimmerman, 2000). However, the multitude of theoretical and psychometric approaches to self-efficacy following Bandura (Chen et al., 2001; Rohner, 2005; Schwarzer & Jerusalem, 1993) has included only limited indications regarding metacognitive dimensions of self-efficacy.

Metacognitive general aspects of self-efficacy refer to what individuals acquire via reflective evaluations of their general, trait-like self-efficacy beliefs; they function as a filter of their general self-efficacy appraisal and monitoring (Nelson & Narens, 1990) that includes metacognitive knowledge and experiences deriving from previous experiences along with the dynamics of information and feeling within the current frame of actions. They encompass beliefs about the self, other people, short- and long-term goals, and life strategies (Kleka et al., 2024). This evaluative metacognitive set of functions, as a filtering process, includes both feelings and evaluative judgments that relate to dealing with difficulties and efforts to adopt strategies for managing long-term important goals (Efklides, 2006); it enables individuals to reorient and recalibrate their strategies on demand and to evaluate outcomes in a more flexible and adaptive manner.

In the Interpersonal Acceptance–Rejection Theory (IPARTheory), personality dispositions as components of psychological maladjustment have been systematically linked to low life satisfaction through restricted adjustment, and they appear as a strong negative predictor of life satisfaction (Rohner & Britner, 2002; Rohner, 2005). Reduced well-being and poor sense of happiness have been strongly associated with emotional instability, hostility, and negative self-view, three out of seven personality dispositions that do not support one’s successful adjustment. This personality sub-theory and the Personality Assessment Questionnaire (PAQ) encompass seven core dispositional dimensions: hostility/aggression, dependence/defensive independence, negative self-esteem, negative self-adequacy, emotional unresponsiveness, emotional instability, and negative worldview. They vary across individuals as a function of their perceived parental acceptance or rejection, thereby predisposing them toward more adaptive or maladaptive patterns of psychological adjustment and, consequently, higher or lower sense of life satisfaction and well-being. Specifically, adolescents’ and young adults’ higher hostility and lower self-esteem correlated with parental rejection were shown to predict moderate to strong levels of lower subjective well-being (Uddin et al., 2014). However, it is important to underline that many relevant studies assessed well-being implicitly and with conceptually equivalent constructs to Diener’s life satisfaction scale (Khaleque & Rohner, 2002; Khaleque & Rohner, 2012).

Well-being and life satisfaction have been found to be enhanced by religiosity. Religiosity is typically treated as a distal personality/value-like variable that influences a meaning and purpose in life and offers a set of coping resources and strategies of everyday life, chances of social networking among religious people, and value consistency based on their faith in God. Individuals with higher religiosity tend to report higher life satisfaction through their positive feelings, a sense of control over their life via faith, and social support they have or expect to have from religious communities (Sujarwoto et al., 2018; Yeniaras & Akarsu, 2017). A cross-cultural study in 29 countries suggested that religiosity moderates positive feelings towards life and life satisfaction as it stabilizes cognitive evaluations of self and restricts emotional fluctuations (Stavrova et al., 2016). Therefore, religiosity is usually modeled as a predictor of the Satisfaction with Life Scale scores. In addition, religiosity and generalized self-efficacy are often conceptualized as parallel psychological resources that enhance perceived control, predictability, and coping confidence. While generalized self-efficacy reflects an internalized belief in one’s personal competence (Chen et al., 2001), religiosity provides a broader meaning system and a sense of God-mediated control that similarly supports coping under uncertainty (Krause, 2005). During the COVID-19 pandemic that brought major disruptions to everyday life worldwide, religiosity appeared to alleviate distress through coping and fear regulation mechanisms and was positively associated with life satisfaction and positive affect through positive cognitive meaning pathways that mitigate stress (Krok et al., 2023; Zacher & Rudolph, 2021). It was also observed that religiosity managed to protect individuals through coping mechanisms of regulating fear but not removing it (Ghoncheh et al., 2021). Generally, empirical findings indicate that even under crisis conditions, religiosity stabilizes global life evaluation and supports individuals’ life satisfaction, and it is mediated by perceived life meaning, stress reduction strategies, and relevant social/spiritual support.

Regarding personal value priorities of the PVQ model, during this pandemic, individuals experienced a decline in values of hedonism and eudaimonic well-being and an increase in self-direction, security, conformity, humility, caring, and universalism, showing that during major societal disruptions, such as a global pandemic, personal values, well-being perceptions, and feelings are flexible and dynamically adapting systems (Bojanowska et al., 2021). Regarding the association between religiosity and personal values, a meta-analysis showed that values of tradition, conformity, and benevolence tend to strengthen the religiosity effects, while self-direction, hedonism, and stimulation tend to weaken it (Saroglou et al., 2004). In general, religiosity seems to align with Conservation and prosocial values, while it is inversely related to autonomy-oriented values. A cross-cultural study suggested that religious individuals prioritize social order and prosocial concern while downplaying autonomy and independent thinking (Schwartz & Huismans, 1995).

The current study aims to explore the interdependence of a system of variables with the individuals’ life satisfaction. This set of variables consists of religiosity, personal values, four personality dispositions that prevent adjustment, and self-efficacy in its general and its metacognitive aspects. These were selected on the basis of previous theoretical approaches, empirical findings, and suggested indications for possibly present mediating effects. To clarify this further, our main aims in this study were to explore for possible mediating effects that PVQ-RR values have on the relationship between personality dispositions, self-efficacy measures, and religiosity on the one hand and life satisfaction on the other hand; in order to be able to test for that, we first had to ascertain that the constructs involved (personal values, personality dispositions, etc., along with their dimensions) existed in our data forming a multivariate system that might be explored further in terms of a structural equation mediation modeling approach.

## 2. Materials and Methods

### 2.1. Sample

The research questionnaire was administered online to 706 Greek adults, all native speakers (511 females, 72.4%, with 4 participants (0.6%) marking “other”). Their mean age was 28.27 (SD = 12.06); although age ranged from 18 to 74 years, the main bulk was 18 to 33 years of age (74.9%), and 468 of the participants (66.29%) were students. Regarding their place of residence, a great majority of the 518 participants (74.10%) reported currently staying in urban locations, 81 participants (11.59%) in suburban locations, and 100 participants (14.31%) in rural locations. Religiosity levels as reported by the 706 participants reached a mean of 4.02 (*SD* = 3.15) on a 10-point scale, with 10 being the most religious point. With respect to religious denomination, 401 participants responded, with 98.3% stating being Christian Orthodox, and with only 7 participants stating differently. With respect to family status, among 705 valid answers, more than half reported being single (53.05%), 21.14 reported being in a relationship without cohabitation, and 11.92% reported living with their partner or under a registered partnership. As expected, due to the over-representation of students (66.29%), only 10.2% were married, and only 3.7% were single due to divorce or the passing of a spouse.

### 2.2. Tools and Procedure

Satisfaction With Life Scale—SWLS (Diener, 1984; Diener et al., 1985). This questionnaire assesses subjective evaluations of the participants’ satisfaction with life. It is based on Diener’s five-item scale assessing a unidimensional sense of life satisfaction (Diener et al., 1985) under a seven-point Likert-type scale (1 = “I strongly disagree” to 7 = “I strongly agree”). The internal consistency (Cronbach’s *a*) for the original Satisfaction with Life Scale is .87 (Diener et al., 1985). The scale exhibits validity in research on individual life satisfaction and also taps additional factors influencing personal judgments, such as current mood and the importance of individuals’ values and life standards (Diener et al., 2013).

Based on the original SWLS, we added three more items specifically created for this study; these addressed the satisfaction derived from relations with family members, intimate relations, and other social relationships. *Example:* “I feel satisfied with my relationships within my family”. A six-point scale was employed in our study following other similar research attempts (Atienza et al., 2003; Blais et al., 1989; Lewis et al., 1999; López-Ortega et al., 2016; Ye, 2007). Cronbach’s *a* index for all eight items in this study was .81.

*Portrait Values Questionnaire, PVQ-RR* (Schwartz et al., 2012). It is designed for Schwartz’s refined theory of basic human values using short verbal portraits of people. Respondents, using a six-point Likert-type scale from 1 = “not like me at all” to 6 = “completely like me”, indicate how similar the person is to themselves.

A previous PVQ model (comprising 54 personal questions) addressed ten distinct original values: self-direction (6 questions), security (6 questions), stimulation (3 questions), conformity (6 questions), hedonism (3 questions), tradition (6 questions), achievement (3 questions), benevolence (6 questions), power (6 questions), and universalism (9 questions). However, the refined version of the scale comprises 57 items (three items per personal value) and addresses 19 values under the *four higher-order values*, as listed next: *Openness to Change* (self-direction in thought, and self-direction in action, stimulation, hedonism) ≠ *Conservation* (tradition, interpersonal conformity, conformity to rules, societal security, personal security); *Self-Enhancement* (achievement, power dominance, power resources, face) ≠ *Self-Transcendence* (universalism tolerance, universalism nature, universalism concern, benevolence dependability, benevolence care, humility.

Two more personal values, humility and face, can be treated as separate personal values, or they may also be included in *Conservation* (Schwartz et al., 2012, 2017). Two examples follow: “This person seeks adventure and likes taking risks” (Stimulation); “Having a good time and enjoying life is important to this person” (Hedonism). Cronbach’s *a,* regarding each of the above 19 personal values, ranged for all 19 values from .67 to .81. Alpha was also calculated for the four higher-order personal values: Self-Transcendence (.81), Self-Enhancement (.67), Openness to Change (.76), and Conservation (.76).

*New General Self-Efficacy scale*, NGSE (Chen et al., 2001). This scale captures how individuals view their capability in meeting task demands within a broad array of contexts (Judge et al., 1998). The NGSE is an eight-item scale, following the Generalized Self-Efficacy scale (GSES), a 10-item scale created earlier and in German by Schwarzer and Jerusalem (1993), assessing the strength of an individuals’ belief in his/her ability to respond to novel or difficult situations and deal with life obstacles, and that too was also based on a previous scale version comprising 14 items (Chen et al., 2000). The NGSE shorter scale is unidimensional and has been shown to be reliable in a sample of students on three occasions—on the first day of class, prior to a midsemester exam, and on the last day of class (*a* = .87, .88, and .85, respectively). The test–retest reliability coefficients of this eight-item scale were high, ranging from .62 to .66. Thus, the eight-item NGSE is a scale that is theory-based, unidimensional, internally consistent, and stable over time (Chen et al., 2001). In our study, Cronbach’s *a* for the NGSE scale reached .91.

*Metacognitive General Self-Efficacy Questionnaire (MGSEQ).* It is a specifically constructed self-report questionnaire (Mrvoljak-Theodoropoulou et al., in press). A total of 841 Greek adults (mean age of 32.04 years) responded to this questionnaire; the majority (56%) were male, and 58.7% (*Ν* = 494) were university graduates. The questionnaire is theoretically framed as a metacognitive measure, but it is important to note that the current item content primarily reflects negatively valenced general self-efficacy beliefs rather than explicit second-order judgments about one’s own general self-efficacy beliefs. However, we used negatively oriented items intentionally in order to alert and motivate respondents to address implicit self-appraisal processes, along with a reflective evaluation of what a person believes about their own restrictive competence. In short, the metacognitive dimension is more strongly grounded at the conceptual level than directly operationalized through explicit item wording. The 10 negatively phrased items regard one’s ability to respond to difficult situations and to deal with obstacles or disappointments without support. Two examples follow: “I usually do not set high goals, due to the fear of failure”, and “In difficult situations it is difficult for me to trust my abilities”. A Likert-type scale from 4 “almost never” to 1 “almost always” anchors “1” as the negative orientation pole. Cronbach’s *a* is as high as .88, and McDonald’s Omega using the Maximum Likelihood estimation model is .88 (Mrvoljak-Theodoropoulou et al., in press). Cronbach’s *a* for the data in the present study is .89.

*Personality Assessment Questionnaire (PAQ Short form).* This is Rohner’s adult version of the Personality Traits Questionnaire, a part of the Interpersonal Acceptance–Rejection Theory (IPARTheory). This personality traits scale is perceived as a psychological maladjustment disposition scale undermining adaptation and well-being. It comprises 63 self-report items grouped into seven distinct subscales: hostility/aggression, dependence, negative self-esteem, negative self-adequacy, emotional responsiveness, emotional instability, and negative worldview (Rohner, 2005). Each of the seven PAQ scales provides an independent measure regarding a theoretically distinct dimension of personality functioning, although “all are interrelated manifestations of overall psychological adjustment” (Rohner & Khaleque, 2005, p. 193). A four-point Likert-type scale is employed from 1 “Almost never true” and 2 “Rarely true” to 3 “Sometimes true” and 4 “Almost always true”, with high scores indicating the negative end. In this study, as we focused on difficulties regarding management of life and the resulting low life satisfaction, we selected four of the seven negative personality dispositions and the respective 36 questions regarding negative self-esteem, negative self-adequacy, emotional instability, and negative worldview; for Rohner’s PAQ theory, these personality dispositions have been shown to be strongly associated with low maladjustment and low well-being. These four specific dispositions were assumed to have a potential association with life satisfaction along with a possible correlation with general self-efficacy under cognitive and metacognitive evaluative processes (Rising & Rohner, 2020). The remaining three personality dispositions regarding perceived experiences of acceptance/rejection were outside the scope of the current study. Cronbach’s *a* for the PAQ generally ranges from .73 to .83 for all seven personality dispositions, with a mean of .81 (Rohner, 2005). In the present study, for the four specific personality dispositions, Cronbach’s *a* ranged from .83 to .90.

All participants completed a Greek version of the questionnaire. It comprised 5 tools (SWLS, NGSE, PAQ short form, MGSE, and PVQ-RR). The first three instruments were subjected to standard back translation procedures; the PVQ-RR instrument had already been translated into Greek for cross-cultural research use (Schwartz et al., 2014). Finally, MGSE was originally constructed in Greek. Religiosity and demographic information were also collected. Religiosity was assessed with just one question, “How religious do you think you are, as a person?”, and it was scored on a 10-point scale from 1 “Not at all” to 10 “Very much”.

The questionnaire required approximately 25–30 min to complete via the Google Forms platform. The link to the form was promoted via social networks, and the participants completed the questionnaire voluntarily and anonymously. This research study complied with the ethical standards and principles pertaining to psychological research. Anonymity was safeguarded by all available means, and data privacy criteria were met.

## 3. Results

Basic descriptive statistics were computed first. Religiosity levels, as reported by the 706 participants, reached a mean of 4.02 (*SD* = 3.15) on a scale of 0 to 10 (with 10 being the most religious). Value scores regarding the four higher-order factor levels, personality dispositions, the general self-efficacy measure, the metacognitive general self-efficacy measure, religiosity levels, and the life satisfaction measure are described in Table 1.

We first attempted some data screening for all available variables by comparing across age bands for possible age-related confounds. Our main aim was to check (also depending on the age band, i.e., younger vs. older adults) whether we might observe differences across tables for two correlation indices, namely, Pearson’s *r* vs. Kendall’s Tau-b. The statistical comparison procedures followed are described in Mylonas et al. (2012) and elsewhere. Under the “Younger” age band condition, differences reached 19%, and under “Older”, they reached 18%. Under no age band condition (overall sample), 22% of differences in correlation pairs across the two index conditions were observed. Then, Kendall’s Tau-b correlations were compared across the two age bands, with no difference found (only approx. 5% of correlation pairs’ differences were observed). Thus, considering age bands or not, we should not trust Pearson’s *r* either for the younger or for the older age band subsamples, or even for the overall sample, and this answered a first important methodological question, with Kendall’s Tau-b correlations to be used in further analysis regarding the data structure.

Confirmatory factor analysis modeling was applied first to test for the four higher-order factors of the 19 PVQ-RR scores alongside their co-existence with several “self” measures (life satisfaction—*LS*, general self-efficacy—*NGSE*, and metacognitive self-efficacy—*MGSE*) and also in connection with four personality disposition measures stemming from IPARTheory (negative self-esteem—*NSE*, negative self-adequacy—*NSA*, emotional instability—*EI*, negative worldview—*NWV*). Following other research examples (e.g., Mylonas et al., 2017, 2023; Zajenkowska et al., 2014), we employed the alternative Tau-b correlation indices for this analysis (as discussed above) to avoid metric bias and achieve a better and clearer depiction of the data potential. In addition, by introducing four error covariances to account for a limited amount of within-factor collinearity (Galanaki et al., 2015), we attempted to confirm the four PVQ-RR higher-order factors (Self-Transcendence—*STr*, Self-Enhancement—*SEnh*, Openness to Change—*OpCh*, and Conservation—*Cons*); in our analysis, these latent traits were expected to accommodate the 19 first-order PVQ-RR factors.

At the same time, we combined all four personality disposition measures (PAQ) into a single construct; the same procedure was followed within the specific CFA model for the three “Self” measures. The model confirmed the structures for the PVQ-RR higher-order set and also for the single constructs for personality dispositions and “Self” measures. The *χ*^2^ criterion was, as always expected, statistically significant (724.544, *df* = 233, *p* < .001), but the *χ*^2^ over *df* criterion was 3.11, which is not far from the desired “2” or less. In addition, the *RMSEA* was 0.055 (95% confidence limits: .050 to .059), and the *SRMR* was .064, *CFI* was .887, and *TLI* (with respect to the null model) was .886; *GFI* was .913, and *AGFI* was .888. Finally, the *χ*^2^ criterion for the difference between the null model and the four-factor one (3907.193, Δ*df* = 43) was statistically significant at the .001 level. These results reassured us that the construct dimensions necessary for the next SEM step in the analysis were indeed present in the data, even if not perfectly modeled. Thus, as the CFA model confirmed the PVQ-RR four-factor structure and also related it with the personality dispositions measures and the “self” measures at an acceptable level, at the next step of the analysis, we attempted mediation modeling regarding personality dispositions measures, value measures, religiosity, general self-efficacy, metacognitive general self-efficacy, and, of course, life satisfaction levels. The correlations analyzed can be found in Table 2.

We expected Rohner’s dimensions (personality dispositions: NSE, NSA, EI, NWV) to be directly related to life satisfaction along with metacognitive self-efficacy, religiosity, and general self-efficacy. We assumed PVQ-RR higher-order values (STr, SEnh, OpCh, Cons) as the mediators in the model. We expected that the personality dispositions (all or some of them) might be mediated through the four PVQ-RR values. In addition, we expected that religiosity, general self-efficacy, and metacognitive general self-efficacy measures’ direct effects on life satisfaction levels might also be mediated through the four PVQ-RR values.

We examined the regression paths first regarding all direct effects in our models, as the statistical assumption dictates that there should exist a statistically significant relation between each of the predictor scores and the predicted one (life satisfaction). This assumption was met, so we could further proceed with our mediation testing. The first attempt showed a very poor fit, leading to the conclusion that personality dispositions would simply remain in the model as direct effect predictors. However, for general self-efficacy and religiosity, there was ample ground for mediation modeling. It was also found through these preliminary attempts that Openness to Change (PVQ-RR) should not remain in our modeling due to possible collinearity with other PVQ-RR higher-order dimensions.

We reached a nearly perfect fit for the finally tested model, with CFI exceeding .90 (.92), and with RMSEA (.039, .16–.062), SRMR (.026), GFI (.993), AGFI (.970), NNFI (.851) all at acceptable levels; as expected, the extremely sensitive *χ*^2^ criterion did not suggest a perfect fit (23.089, *df* = 11, *p* < .05), but the *χ*^2^ over df ratio reached acceptable levels approaching 2 (2.099), and the *χ*^2^ difference with respect to the baseline model was statistically significant (Δχ^2^ with baseline = 152.659, Δ*df* = 10, *p* < .001). Three personality disposition measures were negatively and significantly predicting life satisfaction (−.111, −.106, −.158, respectively, for NSE, NSA, and NWV). Non-significant direct weights towards life satisfaction appeared for the MGSE and the NGSE measures. However, we observed a small but significant direct weight of religiosity on life satisfaction (.069, *p* < .05).

**Figure 1:**
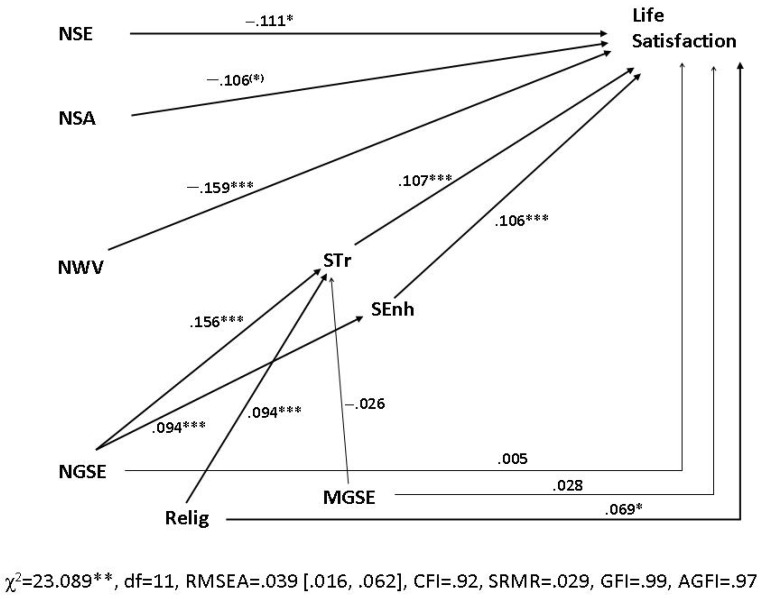
Mediation modeling: an attempt to address life satisfaction through personal values. **Note:** (*) marginally significant, * *p* < .05, ** *p* < .01, *** *p* < .001; non-significant paths appear as finer lines.

A partial mediation effect was detected regarding the religiosity to life satisfaction and the general self-efficacy to life satisfaction direct effects, as mediated through the Self-Transcendence (STr) value, along with general self-efficacy to life satisfaction, as mediated through the Self-Enhancement (SEnh) value (as shown in Figure 1). It seems that the religiosity’s direct effect on life satisfaction is partially inflated when Self-Transcendence mediates the path. In addition, the practically nonexistent direct effect between general self-efficacy and life satisfaction seems to “come to life” when Self-Transcendence and Self-Enhancement are involved; thus, we may deduce that when the combined values detour is active, general self-efficacy becomes more important for life satisfaction. However, the overall indirect effect is a small one (although statistically significant at the .01 level and with confidence limits for the parameter between .003 and .017). This result indicates partial mediation only, so the initial direct effects are only partly changed. The total effect observed (−.101) was statistically significant at the .05 level. The final outcomes for this mediation model are fully presented in Figure 1.

Finally, Sobel tests (Aroian) followed and indicated that the strongest mediation path of the three was the one involving general self-efficacy to Self-Enhancement to life satisfaction, with the other two mediation paths closely following.

## 4. Discussion

The first important outcome of this study is that the four higher-order values were present in our data, namely, Self-Transcendence, Self-Enhancement, Openness to Change, and Conservation. This is a very important finding regarding the value structure in Schwartz’s theory as it verifies the existence of the expected first-order values in their superlative domains. Along with that, our analysis showed that factor analysis can depict these higher-order value dimensions in a data set, although this is not the mainstream analysis followed, with the usual approach being variations of multidimensional scaling. This finding might also be an output of the use of the alternative correlation indices employed (Kendall’s Tau-b), an action that might have released even further potential existing in this data set (Mylonas et al., 2023; Zajenkowska et al., 2014). The four higher-order factors were found to be moderately to highly correlated. The stronger correlations were found between Self-Transcendence and Openness to Change, and Openness to Change and Self-Enhancement. Self-Transcendence was also highly correlated with Conservation, whereas all remaining correlations were at the moderate level. In particular, the Self-Transcendence and Self-Enhancement correlation was even lower (.34). Thus, it seems that Self-Transcendence and Openness to Change are strongly linked, and in turn, Openness to Change is strongly linked to Self-Enhancement, which is not close to Self-Transcendence. Conservation is closer to Self-Transcendence than to any other higher-order value factor.

An additional important finding was that the incorporation of “non-value” measures, including self-efficacy and life satisfaction, contributed significantly to the model’s explanatory power. Furthermore, the relations observed with these measures, and separately within their own domain, excluding personal values, enabled us to consider a broader system of variables in the subsequent mediation modeling. Although the findings regarding this system of measures are in line with previous results (Çakar, 2012; Azizli et al., 2015; Poorbaferani et al., 2018), they also enrich the association of self-efficacy beliefs with life satisfaction by having introduced other aspects of self-efficacy as well (i.e., metacognitive) in the overall model fit.

Regarding the mediation analysis, the meaning of Self-Transcendence and Self-Enhancement values for life satisfaction seems to be highly enriched by the partial mediating role of these higher-order values with respect to the relationship between general self-efficacy and life satisfaction for adults in Greece. The novel contribution is with regard to the study of the influence of these two higher-order personal values alongside the effect that general self-efficacy exerts on life satisfaction. Previous research has highlighted strong positive correlations between certain personal values—such as self-direction, benevolence, universalism, achievement, and stimulation and achievement—and life satisfaction, likely due to their role in fostering personal growth and development. In contrast, values such as security, tradition, and conformity have been shown to correlate negatively with life satisfaction, as they are often associated with self-restriction and a heightened need for self-protection in the face of uncertainty or adversity (Bilsky & Schwartz, 1994; Sagiv & Schwartz, 2000). Regarding the finding of Self-Transcendence and Self-Enhancement values mediating the role of the effects of self-efficacy on life satisfaction, it may lead researchers to reiterate the previous results on the predictive function of self-efficacy on adults’ life satisfaction (Çakar, 2012).

Another important result showed the Self-Transcendence partial mediating role for the relationship between religiosity and life satisfaction. A cross-cultural study showed that religious individuals prioritize social order and prosocial concern, but they do not prioritize autonomy and independent thinking (Schwartz & Huismans, 1995). Other research on how religiosity is strengthened by specific personal values suggested that personal values of tradition, conformity, and benevolence function towards this direction, but values of self-direction, hedonism, and stimulation tend to function towards religiosity weakening (Saroglou et al., 2004). In our analysis, similar fragments of evidence seem to fall into place.

The importance of personal values of Self-Transcendence and Self-Enhancement appeared as key mechanisms through which individuals interpret their capabilities and life experiences to act in reaching overall life satisfaction. These two higher-order personal values seemed to constitute a fundamental framework through which individuals make sense of their beliefs about self-efficacy and their satisfaction in life; however, only Self-Transcendence values, which reflect concern for the welfare of others and nature, were positively interpreted by the participants’ religiosity as ultimately influencing their overall life satisfaction. These findings may contribute to a deeper understanding of how Schwartz’s higher-order values function psychologically. The identification of Self-Transcendence and Self-Enhancement as mediating the predictive relationship of self-efficacy on life satisfaction advances our understanding of their interconnective pathways. Although this specific exploration of the interdependence of self-evaluation variables and these two higher-order personal values is not the only possible one, it manages to reveal a mediating role of Self-Transcendence and Self-Enhancement in the predictive effect of self-evaluation judgments on life satisfaction. This finding may also be interpreted in light of the specific and active gradual social change in Greece, which is likely to have progressively influenced individuals’ personal value orientations, behaviors, and perceptions of life satisfaction. These shifts follow the widespread disruptions to everyday life associated with the COVID-19 pandemic and, in the Greek context, a preceding decade of severe economic crisis (2009–2019) that fundamentally challenged individuals’ sense of life satisfaction.

There were some non-findings as well; these also require our attention. The first striking non-finding was that two of the higher-order value factors, namely, Conservation and Openness to Change, did not exhibit enough potential to participate in our hypothesized mediation model. Even though Conservation was not a very strong mediator candidate, Openness to Change was indeed expected to appear in the model and enhance the relationship between the self-efficacy measures and life satisfaction, if not active further. Such a mediation would signify the acceptance or rejection of rapidly changing parameters of everyday life as parts of the perception of self-efficacy and its relation to life satisfaction. Since in the present Greek setting, adults are faced with continuous changes in their everyday lives, this may have become a burden to some of them but an opportunity to others. Consequently, these two influences may cancel each other, rendering Openness to Change an inert node in the pathway linking self-appraisal of already acquired potential to life satisfaction. Another surprising result was that the four personality dispositions were not mediated by any of the higher-order values, and they were just directly related to life satisfaction. In addition, emotional instability evaded the model. Finally, although our modeling remained at the level where values are the mediators, one might instead expect a reverse sequence with personality dispositions being the mediators (with respect to the relationship of values with life satisfaction). The same might be true for the relationships of the self-efficacy measures with life satisfaction, and for religiosity too. Thus, personality dispositions might be considered possible mediators in some future research modeling, or even better, the model might account for their mediating potential in a recursive manner between these measures and personal values.

Despite the fact that this study is limited by its use of a convenience sample, the research findings offer important initial results for the function of the PVQ-RR higher-order personal values on life satisfaction. This study has not demonstrated how the Openness to Change and Conservation values can shape the links between one’s beliefs about self and sense of life satisfaction and has not clarified the predictive properties of the four personality dispositions, as these may be considered as mediators too. Further research could contribute to the exploration of other additional “mediating variables” that may underlie the interactions between self-evaluation variables and PVQ higher-order personal values in predicting life satisfaction.

### Limitations

As noted above, our sampling was a non-random one but we also have to note that the female population is over-represented, and this may have undesirable effects on the analysis posing an obvious caveat with respect to the generalization power of the study; however, it also hints a future research field as we might address the two genders separately (as described in Table 1) and compare the respective structural models. Another limitation concerns the over-representation of university students (66%), which may restrict the generalizability of the findings. In addition, although most participants were young adults (75%), the broader age range included in the sample may have introduced further variability and limited generalizability; to our defense, using Kendall’s Tau-b indices, after showing no differences across age bands, may be considered a remedy to this problem. The religious denomination distribution, with only seven participants following a different denomination, does not create a similar problem, though, as we measure religiosity and not religious denominations. In all, the social diversity present in our data was not directly addressed and explored as we focused mainly on the structure and the mediating roles of personal values; this social diversity factor may be a target to consider in future research. Of course, apart from all these, other method factors may have blurred our results, such as bias in terms of culture or the fact that we have worked with self-report data, a quite usual shortcoming in psychological research. Finally, the online recruitment of the sample is one more procedural detail to note, as it may be susceptible to self-selection bias.

A separate note should be drawn with respect to the correlation explanatory power, especially when correlational studies include path structures, as our own study did. Many studies focus on the statistical significance that accompanies various correlation indices and disregard or underestimate the magnitude of the index; thus, significance levels (e.g., for Pearson’s r) are highly dependent on the actual N producing the index. A very large sample size will yield statistical significance even for small correlation indices (even as small as .08 or less), an outcome that has no real meaning in psychological science (with r^2^ reaching zero in such cases). A small sample will have the opposite effect (type II error). Beta values are also affected in a similar manner, so one has to be careful with respect to interpretation, as small Beta values indicate small path power, but they may appear significant without explaining things really. Consequently, our findings suggest that personal values play a modest mediating role in the relationship between self-evaluations and sense of religiosity with life satisfaction. The observed effects were relatively small, indicating that personal values account for only a limited part of this association and that additional factors are likely to contribute to the relationship. Future research studies may shed light on such questions.

## 5. Conclusions

The present study provides empirical support for the higher-order structure of Schwartz’s value theory, demonstrating the presence of the four expected higher-order values—Self-Transcendence, Self-Enhancement, Openness to Change, and Conservation—in a Greek adult sample. The findings may further suggest that factor analysis could successfully capture these higher-order value dimensions, offering an alternative methodological approach to the multidimensional scaling techniques more commonly employed in value research. In addition, the incorporation of self-efficacy, life satisfaction, religiosity, and related psychological measures enhanced the explanatory power of the model and enabled a broader examination of the interrelationships among values, self-evaluative beliefs, and life satisfaction. The observed associations were generally consistent with previous literature while extending it through the inclusion of a metacognitive aspect of self-efficacy.

The mediation analyses highlighted the particular importance of Self-Transcendence and Self-Enhancement values in understanding adults’ life satisfaction. Both values partially mediated the relationship between general self-efficacy and life satisfaction, while Self-Transcendence also mediated the relationship between religiosity and life satisfaction. These findings suggest that personal values constitute important psychological mechanisms through which self-evaluations and religious orientations may influence life satisfaction. At the same time, the absence of mediating effects for Openness to Change and Conservation, as well as the direct rather than mediated effects of three personality dispositions—negative self-esteem, negative self-adequacy, and negative worldview—indicates that the interplay among values, personality, self-efficacy, and religiosity is a complex set of parameters that influence life satisfaction.

In conclusion, the study advances understanding of the role of higher-order personal values in life satisfaction by demonstrating that Self-Transcendence and Self-Enhancement personal values function as meaningful mediators linking self-evaluative beliefs and religiosity to life satisfaction. Future research should, therefore, explore alternative and potentially recursive mediation pathways, examine additional mediating variables, and test these relationships in a representative adult sample.

## Figures and Tables

**Table 1:** Descriptive statistics by age band, by gender, and for the total sample.

	Overall Sample N = 706						Age 18–33 *n* = 529	Age 34–74 *n* = 177	Males *n* = 151	Females *n* = 511
	Min.	Max.	Mean	SD	Sk	Kurt	Mean (SD)	Mean (SD)	Mean (SD)	Mean (SD)
STr	1.00	6.00	4.95	.67	−1.33	3.582	4.97 (.64)	4.88 (.76)	4.88 (.67)	4.99 (.66)
SEnh	1.00	6.00	3.57	.88	.09	−.165	3.60 (.87)	3.51 (.91)	3.60 (.93)	3.57 (.86)
OpCh	1.00	6.00	4.73	.68	−.77	1.285	4.78 (.66)	4.59 (.73)	4.69 (.70)	4.76 (.67)
Cons	1.00	5.00	3.40	.73	−.54	.163	3.31 (.74)	3.69 (.62)	3.21 (.79)	3.48 (.69)
NSE	1.00	4.00	1.89	.63	.62	−.252	2.02 (.63)	1.52 (.44)	1.87 (.61)	1.89 (.63)
NSA	1.00	4.00	1.92	.67	.57	−.378	2.06 (.67)	1.50 (.44)	1.88 (.67)	1.92 (.66)
EI	1.00	4.00	2.53	.57	−.12	−.352	2.65 (.55)	2.21 (.50)	2.33 (.60)	2.61 (.54)
NWV	1.00	4.00	2.05	.61	.37	−.234	2.15 (.61)	1.74 (.52)	2.10 (.66)	2.02 (.59)
LS	1.50	6.00	4.30	.77	−.57	.048	4.27 (.78)	4.37 (.75)	4.18 (.82)	4.35 (.74)
MGSE	1.00	4.00	2.54	.67	.01	−.611	2.42 (.66)	2.89 (.57)	2.67 (.69)	2.50 (.65)
NGSE	1.00	5.00	3.81	.67	−.60	.847	3.71 (.69)	4.14 (.49)	3.87 (.69)	3.80 (.66)
Relig	.00	10.00	4.02	3.15	.16	−1.233	3.58 (3.06)	5.33 (3.09)	2.91 (3.13)	4.43 (3.06)

**Key:** STr = Self-Transcendence, SEnh = Self-Enhancement, OpCh = Openness to Change, Cons = Conservation, NSE = negative self-esteem, NSA = negative self-adequacy, EI = emotional instability, NWV = negative worldview, LS = life satisfaction, MGSE = metacognitive general self-efficacy, NGSE = general self-efficacy, Relig = religiosity. **Note 1:** std. error for skewness = .092; std. error for kurtosis = .184. **Note 2:** Age bands were defined according to the third quartile.

**Table 2:** Correlations between all main variables.

	STr	SEnh	OpCh	Cons	NSE	NSA	EI	NWV	LS	MGSE	NGSE	Relig
STr	1.00											
SEnh	.015	1.00										
OpCh	.402	.146	1.00									
Cons	.393	.244	.294	1.00								
NSE	−.076	−.045	−.115	−.142	1.00							
NSA	−.091	−.079	−.149	−.177	.708	1.00						
EI	−.048	−.004	−.072	−.061	.461	.480	1.00					
NWV	−.078	−.050	−.105	−.168	.459	.461	.402	1.00				
LS	.197	.088	.175	.208	−.295	−.306	−.220	−.293	1.00			
MGSE	.053	.037	.110	.060	−.503	−.554	−.543	−.348	.212	1.00		
NSGE	.156	.157	.204	.214	−.470	−.542	−.359	−.336	.224	.490	1.00	
Relig	.051	.136	.035	.349	−.096	−.169	−.029	−.137	.141	.028	.127	1.00

**Key:** STr = Self-Transcendence, SEnh = Self-Enhancement, OpCh = Openness to Change, Cons = Conservation, NSE = negative self-esteem, NSA = negative self-adequacy, EI = emotional instability, NWV = negative worldview, LS = life satisfaction, MGSE = metacognitive general self-efficacy, NGSE = general self-efficacy, Relig = religiosity. **Note:** The indices are Kendall’s Tau-b correlations. The basic descriptive statistics are given in Table 1.

## Data Availability

The data are currently retained at the National and Kapodistrian University of Athens (by agari@psych.uoa.gr), and they are available to the Journal upon request.
